# Association of the Single Nucleotide Polymorphisms in microRNAs 130b, 200b, and 495 with Ischemic Stroke Susceptibility and Post-Stroke Mortality

**DOI:** 10.1371/journal.pone.0162519

**Published:** 2016-09-07

**Authors:** Jinkwon Kim, Gun Ho Choi, Ki Han Ko, Jung Oh Kim, Seung Hun Oh, Young Seok Park, Ok Joon Kim, Nam Keun Kim

**Affiliations:** 1 Department of Neurology, CHA Bundang Medical Center, School of Medicine, CHA University, Seongnam-si, 13496, South Korea; 2 Institute for Clinical Research, CHA Bundang Medical Center, School of Medicine, CHA University, Seongnam-si, 13496, South Korea; 3 Department of Neurosurgery, Chungbuk National University Hospital, Chungbuk National University, Cheongju-si, 28644, South Korea; 4 Department of Biomedical Science, College of Life Science, CHA University, 335 Pangyo-ro, Bundang-gu, Seongnam-si, 13488, South Korea; Institut de Pharmacologie Moleculaire et Cellulaire, FRANCE

## Abstract

The microRNA (miRNA) is a small non-coding RNA molecule that modulates gene expression at the posttranscriptional level. Platelets have a crucial role in both hemostasis and thrombosis, a condition that can occlude a cerebral artery and cause ischemic stroke. *miR*-*130b*, *miR*-*200b*, and *miR*-*495* are potential genetic modulators involving platelet production and activation. We hypothesized that single nucleotide polymorphisms (SNPs) in these miRNAs might potentially contribute to the susceptibility to ischemic stroke and post-stroke mortality. This study included 523 ischemic stroke patients and 400 control subjects. We investigated the association of three miRNA SNPs (*miR*-*130b*T>C, *miR*-*200b*T>C, and *miR*-*495*A>C) with ischemic stroke prevalence and post-stroke mortality. In the multivariate logistic regression, there was no statistically significant difference in the distribution of *miR*-130bT>C, *miR*-*200b*T>C, or *miR-495*A>C between the ischemic stroke and control groups. In the subgroup analysis based on ischemic stroke subtype, the *miR*-200b CC genotype was less frequently found in the large-artery atherosclerosis stroke subtype compared with controls (TT+CT vs CC; adjusted odds ratio for CC, 0.506; 95% confidence interval, 0.265–0.965). During a mean follow-up period of 4.80 ± 2.11 years after stroke onset, there were 106 all-cause deaths among the 523 stroke patients. Multivariate Cox regression analysis did not find a significant association between post-stroke mortality and three miRNA SNPs. Our findings suggest that the functional SNP of *miR*-*200b* might be responsible for the susceptibility to large-artery atherosclerotic stroke.

## Introduction

Stroke is a major public health issue and a leading cause of death and disability worldwide [1]. Stroke is a multifactorial disease influenced by multiple genetic and environmental factors. About 80% of stroke cases have an ischemic origin, and the triggering ischemia is commonly due to atherothrombotic or embolic occlusion of a cerebral artery. Platelets have a crucial role in hemostasis and atherothrombosis, as they are a major component of thrombi [2]. Abnormally increased platelet activity is a predisposing factor to the formation of thrombi and the occurrence of acute vascular diseases such as myocardial infarction and ischemic stroke [3, 4].

MicroRNA (miRNA) is a small endogenous non-coding RNA molecule that modulates protein synthesis by binding to the 3′ untranslated regions of protein-coding gene transcripts (messenger RNA, mRNA). miRNA binding leads to the degradation of the target mRNA, resulting in translational repression [5]. Altered miRNA regulation has been implicated in the pathogenesis of various disorders including stroke. A large number of miRNAs are expressed in platelets, and miRNA expression profiles vary with platelet biogenesis, maturation, and activation [6, 7]. This dynamic miRNA expression in platelets is considered a novel genetic regulatory pathway for platelet formation and activation, and might be an underlying pathway for the pathogenesis of thrombotic disorders [8–10]. In a prior study of the expression pattern of miRNA during platelet production and differentiation, *miR-130b*, *miR-200b*, and *miR-495* were found to be significantly down-regulated during megakaryocyte maturation [11]. *miR-130* targets MAFB, a transcription factor required for promoting platelet development [12–14]. *miR-200b* and *miR-495* knocked down PRKAR2B and KLHL5, respectively, and both are platelet functional proteins [15]. In human platelets, *miR-495* was up-regulated with thrombin stimulation compared with the expression in the resting condition [16].

Single nucleotide polymorphisms (SNPs) are the most frequent type of genetic variation in the human genome. miRNA-related SNPs are defined as SNPs in miRNA coding genes, miRNA target binding sites, miRNA regulatory regions, and miRNA processing machinery [17, 18]. miRNA-related SNPs can influence miRNA functions and target gene expressions [19], and their functional consequences can result in phenotypic variation and a predisposition to various diseases [20, 21]. There is evidence that a miRNA SNP (*miR*-*146a* rs2910164) could contribute to the susceptibility to ischemic stroke [21–23]. *miR-618* SNP (rs2682818) is suggested as a genetic risk marker for ischemic stroke recurrence [24]. However, it is not known whether genetic variations in miRNA associated with platelet physiology have a functional role in the pathogenesis of ischemic stroke.

As mentioned above, *miR*-*130b*, *miR-200b*, and *miR*-*495* have been previously demonstrated to be expressed differently during platelet biogenesis and activation [11, 25]. We focused on three SNPs located in these miRNA regulatory regions: *miR*-*130b*T>C (rs373001), *miR*-*200b*T>C (rs7549819), and *miR*-*495*A>C (rs2281611). The target genes of these miRNAs are involved in the activation, aggregation, and pro-inflammatory reaction of platelets [9, 15, 26–29]. These miRNA SNPs may modulate platelet function. Thus, given the critical role played by platelets in thrombosis and vascular biology, we hypothesized that these miRNA SNPs ultimately influence an individual’s genetic susceptibility to ischemic stroke and post-stroke prognosis. In this study comprising over 900 Korean subjects, we explored the susceptibility to ischemic stroke and mortality after ischemic stroke according to these miRNA SNPs.

## Methods

### Study participants

For this study, 923 individuals (523 patients with ischemic stroke and 400 controls) were enrolled from 2000 to 2008. The study subjects were consecutively recruited from the Department of Neurology at CHA Bundang Medical Center, CHA University (an 800-bed teaching hospital in Gyeonggi-do, South Korea). All recruited subjects underwent medical history evaluation, routine laboratory tests (including a complete blood count and electrocardiogram), and brain magnetic resonance imaging (MRI) or computed tomography (CT). Ischemic stroke was defined as an acute neurological dysfunction of vascular origin confirmed by brain MRI or CT. Based on clinical findings and neuroimaging data, two neurologists classified the ischemic stroke patients into four stroke subtypes using the Trial of Org 10172 in Acute Stroke Treatment (TOAST) criteria as follows: (1) large-artery atherosclerosis (LAA), (2) cardioembolism (CE), (3) small-vessel occlusion (SVO), and (4) other patients with undetermined etiology or incomplete evaluation [30]. Briefly, LAA patients had 50% or greater stenosis of a relevant cerebral artery confirmed by cerebral angiography, SVO patients showed an infarction lesion < 15 mm in diameter and classic lacunar syndrome without evidence of a cerebral cortical dysfunction or other underlying etiology, and CE patients had a cardioembolic source such as atrial fibrillation as detected by cardiac evaluation [30]. The frequencies of the four stroke subtypes were 39.4% (n = 206), 27.0% (n = 141), 10.9% (n = 57), and 22.8% (n = 119) for LAA, SVO, CE, and other, respectively. The 400 control subjects were recruited from individuals who visited our hospital for health examinations. The control subjects did not have a previous history of cerebrovascular disease or myocardial infarction. No significant difference was found in the age and sex distribution between the ischemic stroke and control groups. This study was approved by the Institutional Review Board of CHA Bundang Medical Center and written informed consent was obtained from all participants.

### Clinical characteristics of the study participants

Study participants were assessed for the presence of hypertension (HTN), diabetes mellitus (DM), hyperlipidemia, and current smoking. HTN was diagnosed when systolic blood pressure ≥ 140 mmHg and diastolic blood pressure ≥ 90 mmHg was found after repeated measurements or participants were currently taking antihypertensive medications. Systolic and diastolic blood pressure values were measured after at least 10 minutes of rest in a seated position. DM was defined as a fasting plasma glucose level >126 mg/dL (7.0 mmol/L) or participants taking diabetic medications. Hyperlipidemia was defined as a high fasting serum total cholesterol level (≥ 240 mg/dL) or a history of antihyperlipidemic agent use. Current smoking was defined as patients who reported smoking in the past year. We also collected the following laboratory data: platelet count; serum levels of folate, vitamin B12, total cholesterol, triglycerides, fibrinogen, antithrombin, blood urea nitrogen, and uric acid; prothrombin time, and activated partial thromboplastin time (aPTT).

### Post-stroke mortality

To evaluate the association of miRNA polymorphism with long-term prognosis after ischemic stroke, survival time from stroke onset to death was compared. The dates of death for each stroke patient (n = 523) were ascertained using death certificates from the Korean National Statistical Office. Patients who were alive on December 31, 2013 were censored.

### SNP genotyping

DNA was extracted using the G-DEX blood extraction kit (iNtRON Biotechnology, Inc., Seongnam, South Korea) according to the manufacturer’s instructions. The three miRNA SNPs selected for this study were *miR*-*130b*T>C [rs373001; chromosome 22, 22007426], *miR*-*200b*T>C [rs7549819; chromosome 1, 1165623], and *miR*-*495*A>C [rs2281611; chromosome14, 101033612]. We used polymerase chain reaction-restriction fragment length polymorphism (PCR-RFLP) assays to analyze *miR*-*200b*T>C and *miR*-*495*A>C SNPs. Genotyping of *miR*-*130b*T>C SNP was performed using real-time PCR (RG-3000, Corbett Research, Australia) for allelic discrimination. Primers and TaqMan probes were designed using Primer Express Software (version 2.0) and synthesized and supplied by Applied Biosystems (USA). The reporter dyes used were FAM and JOE. The *miR*-200bT>C SNP was detected using the following forward and reverse primers: 5′- CCT GAA CCT GGC AGT GG -3′ and 5′- CAG TGC TTC AGG AAC ACA ATT T -3′, respectively. The *miR-495*A>C SNP was detected using the following primers to generate a 95-bp product: 5′- GCA TCA GGT AAG TTG GGT CA -3′ (underlined nucleotide was the mismatch sequence) and 5′- TTA TCC GTG ATG ACT GTC CG -3′ for forward and reverse, respectively. The *miR*-*130b*T>C SNP was detected by TaqMan probe assay kit (Life Technologies, Carlsbad, CA, USA). *miR*-*200b*T>C and *miR*-*495*A>C SNPs were digested with 5U *Aci*I and 5U *Hin*cII, respectively, for 16 h at 37°C (New England BioLabs, Beverly, MA, USA). The PCR annealing temperature was 60°C for all SNPs, with 35 cycles (*miR*-*200b*T>C/*miR-495*A>C) or 50 cycles (*miR*-*130b*T>C) for the amplification step in PCR progressions. The PCR product (12 μL) was run on a 3.0% ethidium bromide-stained agarose gel and directly visualized under ultraviolet illumination in *miR*-*200b*T>C and *miR*-*495*A>C, because these were performed using a PCR-RFLP assay. We randomly repeated approximately 10% of the PCR assays for each of the miRNA polymorphisms and checked the results for concordance with DNA sequencing using an automatic sequencer (ABI3730x l DNA analyzer; Applied Biosystems, Foster City, CA). The concordance of the quality control samples was 100%.

### Statistical analyses

To analyze the differences in the clinical characteristics of the study groups, we used Fisher’s exact test for categorical data and independent t-test or one-way analysis of variance for continuous data. We compared the genotype distributions of *miR*-*130b*, *miR*-*200b*, and *miR*-*495* SNPs in ischemic stroke patients and control subjects using binary logistic regression analyses. The association of miRNA SNPs with post-stroke mortality was evaluated using Cox proportional hazard regression. The proportional hazards assumption was tested using a log(-log(survival)) plot and interaction for follow-up time in a time-dependent Cox regression model, which was found to be satisfactory. In the multivariate analyses, adjustments were performed for sex, age, HTN, DM, hyperlipidemia, and current smoking, all of which are well-established risk factors for ischemic stroke. Data were analyzed using GraphPad Prism 4.0 (GraphPad Software Inc., San Diego, CA), Medcalc version 12.7.1.0 (Medcalc Software, Mariakerke, Belgium) and R software, version 3.2.1 for Windows (The R Foundation for Statistical Computing, Vienna, Austria). Allele combinations of multiple loci were analyzed using the expectation-maximization algorithm with SNPAlyze (Version 5.1; DYNACOM Co. Ltd, Yokohama, Japan). We calculated the statistical power using the CaTS Power Calculator (http://csg.sph.umich.edu//abecasis/cats/index.html) [31]. Stroke prevalence is estimated to be 1.59% in Korean adults ≥ 30 years of age [32]. Under the assumption of a dominant model, minor allele frequency of 20%, type I error level of 0.05, and the sample size of this study (400 controls and 523 cases), we had 80% power to detect an association of a SNP with a genetic relative risk of 1.50.

## Results

### Clinical characteristics of the study subjects

The clinical characteristics of the 523 ischemic stroke patients and the 400 controls are summarized in Table 1. There was no statistically significant difference in the age or sex distribution between ischemic stroke patients and controls. However, ischemic stroke patients were more likely to have HTN and DM than control subjects. When we compared the laboratory tests results, increased homocysteine and fibrinogen levels and decreased folate levels and aPTT were found in ischemic stroke patients. Platelet counts did not significantly differ between the two groups.

**Table 1:** Baseline characteristics in ischemic stroke patients and control subjects.

Characteristic		Control	Stroke	*P*^a^
		(n = 400)	(n = 523)	
Risk factors	Sex (male)	171 (42.7)	226 (43.2)	0.952
	Age, year	63.69 ±10.45	64.16 ± 10.36	0.500
	Hypertension	168 (42.0)	342 (65.4)	<0.001
	Diabetes mellitus	54 (13.5)	150 (28.7)	<0.001
	Hyperlipidemia	95 (23.7)	148 (28.3)	0.244
	Current smoking	135 (33.7)	194 (37.1)	0.476
Laboratory findings	Homocysteine, μmol/l	10.13 ± 4.22	11.29 ± 6.62	0.002
	Folate, nmol/l	8.89 ± 8.02	6.95 ± 5.19	<0.001
	Vitamin B12, pg/ml	747.19 ± 673.14	751.85 ± 655.40	0.916
	Total cholesterol, mg/dl	193.39 ± 37.56	189.33 ± 41.60	0.129
	Triglyceride, mg/dl	147.43 ± 90.80	151.33 ± 113.86	0.578
	PLT, x10^3^/μl	242.99 ± 66.12	247.73 ± 87.26	0.368
	PT, sec	11.77 ± 0.78	11.92 ± 3.27	0.413
	aPTT, sec	33.33 ± 18.69	30.54 ± 4.43	0.001
	Fibrinogen, mg/dl	397.47 ± 120.81	431.05 ± 132.01	0.007
	Antithrombin, %	93.81 ± 44.37	93.45 ± 17.01	0.885
	BUN, mg/dl	15.91 ± 5.06	16.32 ± 7.66	0.419
	Uric acid, mg/dl	4.67 ± 1.47	4.67 ± 1.59	0.983

Data are expressed number (%) or mean ± standard deviation. PLT, platelet; PT, prothrombin time; aPTT, activated partial thromboplastin time; BUN, blood urea nitrogen.

^a^P-values were calculated using a two-sided t-test for continuous variables and Fisher`s exact test for categorical variables.

### miRNA SNPs genotype and risk of ischemic stroke

We investigated the distributions of *miR*-*130b*T>C, *miR*-*200b*T>C, and *miR*-*495*A>C SNPs in ischemic stroke patients and controls. The frequencies of miRNA genotypes in control subjects were consistent with the expected frequencies under Hardy-Weinberg equilibrium. Table 2 shows the results of the multivariate logistic regression analyses adjusted for sex, age, HTN, DM, hyperlipidemia, and current smoking. There was no statistically significant difference in the distribution of *miR*-*130b*T>C, *miR*-*200b*T>C, or *miR*-*495*A>C SNPs between ischemic stroke patients and controls. Individual participant's genotype and clinical information are shown in S1 Table.

**Table 2:** Genotype frequencies of microRNA gene polymorphisms in controls and ischemic stroke patients.

SNP	Genotype	Control (n = 400)	Stroke (n = 523)	AOR (95% CI)	*P*	*FDR-P*
***miR*-*130b*T>C rs373001**	TT	228 (57.0)	290 (55.4)	1.000 (reference)		
	TC	144 (36.0)	192 (36.7)	1.021 (0.764–1.365)	0.887	0.887
	CC	28 (7.0)	41 (7.8)	1.168 (0.686–1.989)	0.568	0.852
	Dominant (TT vs TC+CC)			1.047 (0.795–1.378)	0.745	0.745
	Recessive (TT+TC vs CC)			1.173 (0.698–1.972)	0.548	0.548
***miR-200b*T>C rs7549819**	TT	174 (43.5)	238 (45.5)	1.000 (reference)		
	TC	177 (44.3)	236 (45.1)	0.976 (0.731–1.302)	0.867	0.887
	CC	49 (12.3)	49 (9.4)	0.748 (0.470–1.191)	0.221	0.663
	Dominant (TT vs TC+CC)			0.929 (0.706–1.222)	0.599	0.745
	Recessive (TT+TC vs CC)			0.762 (0.491–1.182)	0.224	0.548
***miR*-*495*A>C rs2281611**	AA	102 (25.5)	123 (23.5)	1.000 (reference)		
	AC	196 (49.0)	280 (53.5)	1.116 (0.798–1.560)	0.523	0.887
	CC	102 (25.5)	120 (22.9)	0.969 (0.654–1.435)	0.875	0.875
	Dominant (AA vs AC+CC)			1.065 (0.776–1.462)	0.697	0.745
	Recessive (AA+AC vs CC)			0.895 (0.651–1.231)	0.496	0.548

Data are derived by multivariate logistic regression adjusted for age, sex, hypertension, diabetes mellitus, hyperlipidemia, and current smoking. AOR = adjusted odds ratio, CI = confidence interval, FDR = false discovered rate, SNP = single nucleotide polymorphism.

#### Subgroup analysis according to ischemic stroke subtype

Because stroke is a heterogeneous disease, the association with miRNA SNPs might be limited to a specific subtype of ischemic stroke. Therefore, we examined the association of the three miRNA SNPs with each ischemic stroke subtype (Table 3). We observed that *miR*-*200b* CC genotype was less frequent in LAA subtype patients than in controls (TT+TC vs CC; adjusted odds ratio (OR) for CC, 0.506; 95% confidence interval (CI), 0.265–0.965; P = 0.039). However, the statistical significance was lost after correcting for multiple comparisons using the false discovery rate method (P = 0.117). No statistically significant difference was found in the distribution of the other miRNA SNPs between stroke subtypes and the control group.

**Table 3:** Comparison of genotype frequencies of microRNA polymorphisms between ischemic stroke subtype and control.

SNP	Genotype	Control (n = 400)	Control vs LAA subtype				Control vs SVO subtype				Control vs CE			
			N (%)	AOR (95% CI)^a^	*P* ^a^	*FDR*	N (%)	AOR (95% CI)^b^	*P* ^b^	*FDR*	N (%)	AOR (95% CI)^c^	*P* ^c^	*FDR*
***miR*-*130b*T>C**	TT	228 (57.0)	123 (59.7)	1.000 (reference)			74 (52.5)	1.000 (reference)			29 (50.9)	1.000 (reference)		
	TC	144 (36.0)	67 (32.5)	0.864 (0.588–1.268)	0.454	0.858	55 (39.0)	1.165 (0.759–1.787)	0.486	0.731	25 (43.9)	1.327 (0.741–2.373)	0.341	0.341
	CC	28 (7.0)	16 (7.8)	1.213 (0.611–2.406)	0.582	0.622	12 (8.5)	1.249 (0.582–2.682)	0.568	0.740	3 (5.3)	0.932 (0.260–3.348)	0.915	0.915
	Dominant (TT vs TC+CC)			0.918 (0.640–1.315)	0.639	0.742		1.172 (0.782–1.756)	0.444	0.771		1.271 (0.724–2.233)	0.404	0.404
	Recessive (TT+TC vs CC)			1.299 (0.664–2.542)	0.445	0.668		1.186 (0.563–2.500)	0.653	0.921		0.828 (0.240–2.856)	0.765	0.765
***miR*-*200b*T>C**	TT	174 (43.5)	95 (46.1)	1.000 (reference)			62 (44.0)	1.000 (reference)			21 (36.8)	1.000 (reference)		
	TC	177 (44.3)	97 (47.1)	0.967 (0.668–1.399)	0.858	0.858	60 (42.6)	0.969 (0.626–1.500)	0.887	0.887	31 (54.4)	1.434 (0.783–2.626)	0.244	0.341
	CC	49 (12.3)	14 (6.8)	0.475 (0.239–0.944)	0.034	0.102	19 (13.5)	1.311 (0.693–2.477)	0.405	0.740	5 (8.8)	0.811 (0.286–2.298)	0.693	0.915
	Dominant (TT vs TC+CC)			0.858 (0.600–1.226)	0.400	0.742		1.038 (0.691–1.560)	0.856	0.856		1.297 (0.725–2.321)	0.382	0.404
	Recessive (TT+TC vs CC)			0.506 (0.265–0.965)	0.039	0.117		1.342 (0.741–2.429)	0.332	0.921		0.701 (0.265–1.856)	0.474	0.711
***miR*-*495A*>C**	AA	102 (25.5)	49 (23.8)	1.000 (reference)			30 (21.3)	1.000 (reference)			10 (17.5)	1.000 (reference)		
	AC	196 (49.0)	104 (50.5)	1.051 (0.676–1.633)	0.827	0.858	78 (55.3)	1.197 (0.721–1.987)	0.487	0.731	35 (61.4)	1.698 (0.795–3.627)	0.172	0.341
	CC	102 (25.5)	53 (25.7)	1.139 (0.678–1.913)	0.622	0.622	33 (23.4)	1.105 (0.614–1.988)	0.740	0.740	12 (21.1)	1.074 (0.427–2.703)	0.880	0.915
	Dominant (AA vs AC+CC)			1.073 (0.707–1.627)	0.742	0.742		1.174 (0.725–1.903)	0.514	0.771		1.453 (0.699–3.022)	0.317	0.404
	Recessive (AA+AC vs CC)			1.075 (0.715–1.614)	0.729	0.729		0.976 (0.610–1.564)	0.921	0.921		0.717 (0.360–1.428)	0.344	0.711

Data are number (%), AOR (95% CI), and P-value for the genotype of microRNA polymorphism. Adjustments were performed for age, sex, hypertension, diabetes mellitus, hyperlipidemia, and current smoking. AOR = adjusted odds ratio, CI = confidence interval, LAA = large-artery atherosclerosis, SNP = single nucleotide polymorphism, SVO = small-vessel occlusion, CE = cardioembolism, FDR = false discovered rate.

^a^ statistics for LAA compared with controls.

^b^ statistics for SVO compared with controls.

^c^ statistics for CE compared with controls.

#### miRNA SNP combination analysis

Next, we conducted possible allele combinations of miRNA to evaluate the combined effect of the polymorphisms in *miR*-*130b*T>C, *miR*-*200b*T>C, and *miR*-*495*A>C on susceptibility to ischemic stroke (Table 4). No allele combination of miRNA reached statistical significance in the analysis of the association between ischemic stroke and ischemic stroke subtype.

**Table 4:** Allele combination analysis of microRNA polymorphisms in ischemic stroke patients and controls.

Allele combination		Control	Stroke	Control vs Stroke			LAA	Control vs LAA subtype			SVO	Control vs SVO subtype		
		(n = 400)	(n = 523)	AOR (95% CI)^a^	*P* ^a^	*FDR-P* ^a^	(n = 206)	AOR (95% CI)^b^	*P* ^b^	*FDR-P* ^b^	(n = 141)	AOR (95% CI)^c^	*P* ^c^	*FDR-P* ^c^
***miR-130b/200b/495***	T-T-A	0.257	0.261	1.000 (reference)			0.260	1.000 (reference)			0.233	1.000 (reference)		
	T-T-C	0.248	0.235	0.896 (0.682–1.176)	0.427	0.744	0.265	1.056 (0.748–1.491)	0.755	0.881	0.224	0.950 (0.623–1.448)	0.810	0.946
	T-C-A	0.107	0.126	0.994 (0.676–1.462)	0.977	0.977	0.117	0.741 (0.432–1.269)	0.275	0.751	0.121	1.357 (0.784–2.348)	0.275	0.868
	T-C-C	0.138	0.117	0.855 (0.625–1.170)	0.327	0.744	0.117	0.908 (0.604–1.364)	0.642	0.881	0.142	1.016 (0.639–1.617)	0.946	0.946
	C-T-A	0.091	0.072	0.865 (0.548–1.363)	0.531	0.744	0.069	0.901 (0.489–1.657)	0.736	0.881	0.090	0.960 (0.485–1.900)	0.906	0.946
	C-T-C	0.060	0.113	1.368 (0.925–2.024)	0.117	0.744	0.103	1.292 (0.778–2.147)	0.322	0.751	0.106	1.482 (0.850–2.587)	0.166	0.868
	C-C-A	0.045	0.045	0.791 (0.451–1.388)	0.414	0.744	0.044	1.011 (0.504–2.031)	0.975	0.975	0.045	0.729 (0.293–1.813)	0.496	0.868
	C-C-C	0.054	0.032	0.926 (0.650–1.320)	0.672	0.784	0.025	0.766 (0.469–1.249)	0.285	0.751	0.040	1.249 (0.749–2.081)	0.395	0.868
***miR-130b/200b***	T-T	0.504	0.497	1.000 (reference)			0.527	1.000 (reference)			0.457	1.000 (reference)		
	T-C	0.246	0.241	0.942 (0.734–1.208)	0.637	0.637	0.233	0.822 (0.591–1.143)	0.244	0.450	0.263	1.185 (0.822–1.709)	0.362	0.533
	C-T	0.152	0.184	1.178 (0.873–1.591)	0.283	0.637	0.170	1.096 (0.742–1.619)	0.646	0.648	0.195	1.289 (0.831–1.999)	0.256	0.533
	C-C	0.098	0.078	0.923 (0.684–1.247)	0.602	0.637	0.070	0.809 (0.542–1.207)	0.300	0.450	0.085	1.153 (0.738–1.801)	0.533	0.533
***miR-130b/495***	T-A	0.364	0.385	1.000 (reference)			0.377	1.000 (reference)			0.354	1.000 (reference)		
	T-C	0.386	0.353	0.883 (0.705–1.105)	0.276	0.486	0.383	1.058 (0.790–1.416)	0.705	0.951	0.366	0.908 (0.649–1.271)	0.575	0.575
	C-A	0.136	0.118	0.832 (0.577–1.199)	0.324	0.486	0.114	0.985 (0.611–1.587)	0.951	0.951	0.136	0.815 (0.469–1.416)	0.467	0.575
	C-C	0.114	0.144	1.099 (0.832–1.451)	0.509	0.509	0.127	1.036 (0.712–1.509)	0.852	0.951	0.145	1.227 (0.825–1.823)	0.312	0.575
***miR-200b/495***	T-A	0.348	0.333	1.000 (reference)			0.327	1.000 (reference)			0.326	1.000 (reference)		
	T-C	0.308	0.348	1.027 (0.808–1.306)	0.827	0.827	0.370	1.127 (0.829–1.532)	0.445	0.445	0.327	1.082 (0.754–1.553)	0.669	0.669
	C-A	0.152	0.170	0.948 (0.681–1.320)	0.753	0.827	0.163	0.835 (0.536–1.299)	0.423	0.445	0.164	1.130 (0.699–1.827)	0.618	0.669
	C-C	0.192	0.150	0.903 (0.700–1.165)	0.434	0.827	0.140	0.867 (0.618–1.215)	0.406	0.445	0.184	1.100 (0.759–1.594)	0.616	0.669

Data are prevalence, AOR (95% CI), and P-value for each allele combination of microRNA polymorphism. Adjustment was performed for sex, age, hypertension, diabetes mellitus, hyperlipidemia, and current smoking. Results for the cardioembolism subtype were omitted due to the small sample size and non-significant finding. AOR = adjusted odds ratio, CI = confidence interval, LAA = large-artery atherosclerosis, SVO = small-vessel occlusion, OR = odds ratio, FDR = false discovered rate.

^a^ statistics for ischemic stroke compared with controls.

^b^ statistics for LAA compared with controls.

^c^ statistics for SVO compared with controls.

#### Interactions of miRNA SNPs with stroke risk factors and laboratory data

To investigate the association of miRNA SNPs in subgroups with specific stroke risk factors, we performed stratified analyses according to sex, age, HTN, DM, hyperlipidemia, and current smoking (S2 Table). When we subdivided participants into two subgroups according to age (median, 63 years), the association between *miR*-*495* AC+CC genotypes (vs AA) and ischemic stroke was statistically significant only in the younger subgroup (adjusted OR, 1.676; 95% CI, 1.011–2.779; P = 0.045). We also evaluated the possible correlations between miRNA SNPs and other laboratory data including platelet count and hematologic findings (S3 and S4 Tables). The only statistically significant relationship was found between the *miR-495* genotypes and level of uric acid (P = 0.034).

### miRNA SNPs with post-stroke mortality

To evaluate the association between miRNA SNPs and post-stroke mortality, Cox regression analysis was performed on the 523 patients with ischemic stroke. During a mean follow-up of 4.80 ± 2.11 years, 106 patients died. In the multivariate Cox proportional hazard regression models, there was no significant association between the three miRNA SNPs and overall survival of patients with ischemic stroke (Table 5). We also did not find a significant association in the subgroup analysis considering ischemic stroke subtypes (Table 6). Allele combination analysis failed to reveal a significant allele combination associated with post-stroke mortality (Table 7).

**Table 5:** Multivariate Cox regression analysis according to miRNA polymorphisms in ischemic stroke patients.

SNP	Genotype	Alive (n = 417, %)	Death (n = 106, %)	Adjusted HR (95% CI)	*P*
***miR-130b*T>C rs373001**	TT	227 (54.4)	63 (59.4)	1.000 (reference)	
	TC	157 (37.6)	35 (33.0)	0.797 (0.525–1.210)	0.287
	CC	33 (7.9)	8 (7.5)	0.932 (0.443–1.960)	0.852
	Dominant (TT vs TC+CC)			0.819 (0.554–1.212)	0.319
	Recessive (TT+TC vs CC)			1.016 (0.491–2.106)	0.965
***miR-200b*T>C rs7549819**	TT	190 (45.6)	48 (45.3)	1.000 (reference)	
	TC	186 (44.6)	50 (47.2)	0.998 (0.669–1.490)	0.993
	CC	190 (45.6)	8 (7.5)	0.875 (0.412–1.858)	0.728
	Dominant (TT vs TC+CC)			0.979 (0.665–1.440)	0.914
	Recessive (TT+TC vs CC)			0.876 (0.424–1.809)	0.720
***miR-495*A>C rs2281611**	AA	93 (22.3)	30 (28.3)	1.000 (reference)	
	AC	230 (55.2)	50 (47.2)	0.842 (0.669–1.490)	0.467
	CC	94 (22.5)	26 (24.5)	0.940 (0.412–1.858)	0.821
	Dominant (AA vs AC+CC)			0.872 (0.564–1.348)	0.538
	Recessive (AA+AC vs CC)			1.054 (0.673–1.649)	0.819

Data are derived by multivariate Cox regression adjusted for age, sex, hypertension, diabetes mellitus, hyperlipidemia, and current smoking. CI = confidence interval, HR = hazard ratio, SNP = single nucleotide polymorphism.

**Table 6:** microRNA polymorphisms and post-stroke survival in each subtype of ischemic stroke.

SNP	Genotype	LAD (n = 206)		SVO (n = 141)		CE (n = 57)	
		Adjusted HR (95% CI)	*P*	Adjusted HR (95% CI)	*P*	Adjusted HR (95% CI)	*P*
***miR*-*130b*T>C rs373001**	TT	1.000 (reference)		1.000 (reference)		1.000 (reference)	
	TC	1.329 (0.705–2.505)	0.379	0.380 (0.105–1.372)	0.140	0.778 (0.273–2.221)	0.699
	CC	1.827 (0.680–4.907)	0.232	0.514 (0.098–2.699)	0.432	<0.001 (NA)	>0.999
	Dominant (TT vs TC+CC)	1.415 (0.783–2.558)	0.25	0.416 (0.133–1.302)	0.132	0.731 (0.256–2.088)	0.558
	Recessive (TT+TC vs CC)	1.631 (0.630–4.221)	0.313	0.760 (0.157–3.683)	0.733	<0.001 (NA)	>0.999
***miR*-*200b*T>C rs7549819**	TT	1.000 (reference)		1.000 (reference)		1.000 (reference)	
	TC	0.891 (0.484–1.642)	0.712	0.894 (0.292–2.738)	0.845	0.555 (0.202–1.526)	0.254
	CC	0.847 (0.245–2.934)	0.793	0.976 (0.183–5.217)	0.977	10.30 (0.614–172.8)	0.105
	Dominant (TT vs TC+CC)	0.886 (0.490–1.599)	0.687	0.912 (0.318–2.612)	0.863	0.658 (0.246–1.761)	0.405
	Recessive (TT+TC vs CC)	0.897 (0.268–2.997)	0.859	1.032 (0.211–5.039)	0.969	0.746 (0.749–201.0)	0.079
***miR*-*495*A>C rs2281611**	AA	1.000 (reference)		1.000 (reference)		1.000 (reference)	
	AC	0.489 (0.237–1.010)	0.053	0.968 (0.251–3.728)	0.962	0.961 (0.165–5.592)	0.965
	CC	0.607 (0.275–1.340)	0.217	1.320 (0.270–6.449)	0.731	0.046 (0.098–4.007)	0.624
	Dominant (AA vs AC+CC)	0.531 (0.274–1.030)	0.061	1.045 (0.286–3.818)	0.948	0.805 (0.146–4.458)	0.804
	Recessive (AA+AC vs CC)	0.978 (0.506–1.892)	0.948	1.354 (0.410–4.468)	0.619	0.651 (0.219–1.936)	0.440

Data are derived by multivariate Cox regression adjusted for age, sex, hypertension, diabetes mellitus, hyperlipidemia, and current smoking. CI = confidence interval, HR = hazard ratio, LAD = large-artery disease, SNP = single nucleotide polymorphism, SVO = small-vessel occlusion, CE = cardioembolism, NA = not applicable.

**Table 7:** Allele combination analysis for the association between microRNA polymorphisms and post-stroke mortality.

Allele combination		Alive (n = 417)	Death (n = 106)	Total stroke (n = 523)		LAD (n = 206)		SVO (n = 141)		CE (n = 57)	
				Adjusted HR (95% CI)	*P*	Adjusted HR (95% CI)	*P*	Adjusted HR (95% CI)	*P*	Adjusted HR (95% CI)	*P*
***miR*-*130b/200b/495***	T-T-A	0.347	0.363	1.000 (reference)		1.000 (reference)		1.000 (reference)		1.000 (reference)	
	T-T-C	0.185	0.208	1.081 (0.743–1.574)	0.684	0.823 (0.454–1.491)	0.520	1.977 (0.655–5.968)	0.227	0.748 (0.306–1.828)	0.524
	T-C-A	0.078	0.075	0.963 (0.561–1.654)	0.891	1.693 (0.725–3.949)	0.224	1.530 (0.409–5.722)	0.527	6.062 (0.342–107.426)	0.219
	T-C-C	0.124	0.113	0.903 (0.568–1.435)	0.665	0.568 (0.259–1.244)	0.157	1.566 (0.414–5.918)	0.508	1.047 (0.404–2.714)	0.925
	C-T-A	0.049	0.033	0.717 (0.327–1.571)	0.406	1.309 (0.445–3.851)	0.625	2.032 (0.349–11.812)	0.430	NA	0.977
	C-T-C	0.098	0.085	0.895 (0.532–1.507)	0.677	1.305 (0.649–2.622)	0.455	0.795 (0.206–3.066)	0.739	2.006 (0.352–11.426)	0.433
	C-C-A	0.025	0.047	1.446 (0.717–2.917)	0.303	1.527 (0.491–4.746)	0.465	1.155 (0.152–8.765)	0.889	1.987 (0.194–20.367)	0.563
	C-C-C	0.095	0.075	0.865 (0.502–1.490)	0.601	1.167 (0.537–2.537)	0.697	0.552 (0.063–4.832)	0.592	0.437 (0.129–1.486)	0.185
***miR*-*130b/200b***	T-T	0.531	0.571	1.000 (reference)		1.000 (reference)		1.000 (reference)		1.000 (reference)	
	T-C	0.201	0.189	0.936 (0.654–1.340)	0.718	0.877 (0.487–1.579)	0.662	0.869 (0.337–2.242)	0.771	1.191 (0.505–2.810)	0.689
	C-T	0.147	0.118	0.794 (0.515–1.225)	0.297	1.357 (0.759–2.427)	0.303	0.743 (0.263–2.101)	0.576	1.240 (0.263–5.849)	0.786
	C-C	0.120	0.123	0.932 (0.608–1.428)	0.745	1.264 (0.666–2.402)	0.474	0.534 (0.145–1.961)	0.344	0.658 (0.244–1.777)	0.409
***miR*-*130b/495***	T-A	0.424	0.439	1.000 (reference)		1.000 (reference)		1.000 (reference)		1.000 (reference)	
	T-C	0.308	0.321	1.014 (0.740–1.390)	0.930	0.669 (0.405–1.107)	0.118	1.622 (0.683–3.857)	0.273	0.832 (0.403–1.719)	0.620
	C-A	0.074	0.080	1.047 (0.614–1.786)	0.866	1.329 (0.589–3.001)	0.493	1.417 (0.375–5.357)	0.607	0.699 (0.076–6.448)	0.752
	C-C	0.193	0.160	0.877 (0.590–1.303)	0.516	1.194 (0.686–2.079)	0.530	0.575 (0.183–1.807)	0.897	0.606 (0.216–1.702)	0.342
***miR*-*200b/495***	T-A	0.396	0.396	1.000 (reference)		1.000 (reference)		1.000 (reference)		1.000 (reference)	
	T-C	0.283	0.292	1.052 (0.754–1.469)	0.765	0.966 (0.584–1.596)	0.891	1.455 (0.593–3.573)	0.413	0.909 (0.393–2.105)	0.824
	C-A	0.103	0.123	1.149 (0.737–1.790)	0.541	1.665 (0.814–3.405)	0.163	1.444 (0.498–4.190)	0.499	3.231 (0.590–17.682)	0.176
	C-C	0.218	0.189	0.908 (0.621–1.327)	0.619	0.741 (0.411–1.336)	0.318	1.096 (0.358–3.358)	0.872	0.812 (0.362–1.819)	0.612

Data are derived by multivariate Cox regression adjusted for age, sex, hypertension, diabetes mellitus, hyperlipidemia, and current smoking. CI = confidence interval, HR = hazard ratio, LAD = large-artery disease, SNP = single nucleotide polymorphism, SVO = small-vessel occlusion, CE = cardioembolism, NA = not applicable.

## Discussion

In this study, we evaluated the association of three miRNA SNPs with susceptibility to ischemic stroke and post-stroke mortality in Korean subjects. *miR*-*130b*, *miR*-*200b*, and *miR*-*495* are potential modulators of platelet production and activity [8, 9]. The *miR*-*130b*T>C (rs373001), *miR*-*200b*T>C (rs7549819), and *miR*-*495*A>C (rs2281611) are regulatory miRNA SNPs located in the promoter regions of miRNA genes. Functional SNPs in miRNA promoters can affect the expression of mature miRNAs, which in turn can have a great impact on miRNA target genes [19, 33]. We did not find a statistically significant difference in the distribution of *miR*-*130b*T>C, *miR*-*200b*T>C, or *miR*-*495*A>C SNPs between ischemic stroke patients and the control group. However, the *miR*-*200b* TT+TC genotype (vs CC) was significantly associated with the LAA stroke subtype. Increasing evidence shows that miRNA-related genetic variance can affect an individual’s predisposition to various diseases and therefore can be a possible underlying pathogenic mechanism [19]. Our findings suggest that the functional SNP of *miR*-*200b* might be responsible for individual differences in the susceptibility to ischemic stroke, the main atherothrombotic disease. In addition, we found that miRNA SNPs were not associated with post-stroke mortality.

Platelets are an essential component of hemostasis and thrombosis. They also play an important role in inflammation, endothelium integrity, and various aspects of the atherothrombotic and atherosclerotic processes. Reduced platelet count and function cause excessive bleeding, whereas increased platelet production and reactivity can contribute to pathologic thrombosis and atherothrombotic diseases including myocardial infarction and ischemic stroke [34, 35]. Therefore, genetic variations in the miRNAs involved in platelet function might be implicated in the pathogenesis of ischemic stroke. In the subgroup analysis of ischemic stroke subtypes, the difference of the genotype distribution in *miR*-*200b*T>C SNP was only significant in the LAA subtype. This subtype-specific association might reflect that platelet activation is a critical pathway in LAA stroke compared with the other stroke subtypes. Platelets play a key role in the transformation of stable atherosclerotic plaques into unstable lesions, which is an important mechanism in LAA stroke, leading to thrombosis and acute ischemic events [2, 36].

Since the first report regarding the role of miRNAs in platelets, the understanding of the mechanisms regulating miRNAs and their modulation of platelet biogenesis and activation has become an expanding research field [7]. Numerous miRNAs have a functional role in multiple steps of platelet biogenesis, including hematopoietic lineage commitment, differentiation, proliferation, maturation, and release into the circulation [11]. Although platelets lack a nucleus, they express abundant pre- and mature miRNAs [9, 37]. Platelets also contain Dicer and Argonaute 2, two protein complexes that are part of the miRNA regulation machinery [6, 38]. By modulating protein synthesis, these platelet miRNAs can have a variety of effects on a platelet’s physiology (e.g., shape change, activation, granule secretion, interaction with the endothelium, vessel damage repair, inflammation, and pro-atherosclerotic effects) [11, 39, 40]. Increasing amounts of data have demonstrated important mechanistic roles for platelet miRNAs in hemostasis, thrombosis, coronary artery disease, and stroke [10, 41]. Therefore, platelet miRNAs are implicated in the pathogenesis of atherothrombotic and cardiovascular diseases [37]. Recently, the possibility of using miRNAs as therapeutic tools in the treatment of cardiovascular diseases has emerged, based on the potential of miRNAs to manipulate the pathologic mechanisms underlying such diseases [39, 42].

In this study, we could not find a significant association between the three SNPs and susceptibility for ischemic stroke except for *miR-200b* and the LAA stroke subtype. However, there are data supporting dynamic roles for *miR*-*130b*, *miR*-*200b*, and *miR*-*495* in platelet function and atherothrombotic diseases. *miR*-*130b* targets *MAFB*, a transcription factor gene that is critical for platelet development and maturation [14, 43]. *miR*-*130b* also targets the peroxisome proliferator-activated receptor gamma (PPARγ), a ligand-activated transcription factor and member of the nuclear hormone receptor protein family that is highly expressed in platelets [28]. PPARγ has a noteworthy hemostatic function: it inhibits the release of soluble CD40 ligand and thromboxane A2, leading to platelet inactivation and vasodilatation [26]. Phosphatase and tensin homologue (*PTEN*) and cytochrome P450 2C9 (*CYP2C9*) are other *miR*-*130b* target genes that are involved in collagen-induced platelet activation and antiplatelet metabolism, respectively [28, 29, 44]. In a genome-wide association study, *miR*-*130b*T>C (rs373001) was found to be associated with myocardial infarction and sudden cardiac arrest in patients with coronary artery disease [45]. *miR*-*200b* targets a regulatory subunit of protein kinase A (*PRKAR2B*) involved in the suppression of platelet activation and aggregation [9, 15]. *miR-495* targets kelch-like family member 5 (*KLHL5*), a gene involved in platelet microtubule and cytoskeleton organization which play a critical role in platelet activation and aggregation [9]. The expression level of *miR*-*495* is positively associated with human platelet reactivity [15, 16]. According to the results obtained with a miRNA target prediction program (TargetScanHuman, available at: http://www.targetscan.org/ Accessed December 15, 2015), the three miRNAs analyzed in this study have potential binding affinities to multiple genes, including *MYB*, *ST18*, *DICER1*, *RUNX1*, and *CDKs*, all of which have a major role in platelet production and activation [25]. We believe that the involvement of miRNA SNPs in platelet biology and its effect on the development and pathogenesis of stroke requires further studies.

Finally, we would like to comment on a few study limitations. This study included only Korean individuals who visited the CHA Bundang Medical Center. The control subjects consisted of individuals recruited when visiting our hospital for health examinations; therefore, the control subjects may not be representative of a general healthy population. We did not perform any external validation and there is the possibility of selection bias. The modest sample sizes of the various subgroups in the subgroup analyses might limit the statistical power of the tests. Except routine hematologic laboratory findings, we did not directly compare functional platelet activity between different miRNA polymorphisms. Due to the limitations of the cross-sectional design, we could not ascertain any causality between the three selected functional miRNA SNPs and the pathogenesis of ischemic stroke. Further studies are needed to evaluate the novel genetic regulatory function of miRNAs on platelet biology and the pathogenesis of stroke.

## Supporting Information

S1 Table Clinical characteristics and genotype of individual participants. (XLS)

S2 Table Association of microRNA genotypes with ischemic stroke susceptibility in subgroup analysis considering individual risk factors. Data are presented as AOR (95% CI) and P-value derived from multivariate logistic regression. Adjustments were performed for age, sex, hypertension, diabetes mellitus, hyperlipidemia, and current smoking. AOR = adjusted odds ratio for ischemic stroke, 95% CI = 95% confidence interval.
(PDF)

S3 Table Differences of various clinical parameters according to microRNA gene polymorphisms in ischemic stroke patients. Data are as mean ± standard deviation and P-value derived from a one-way analysis of variance test. PLT = platelet count, PT = prothrombin time.
(PDF)

S4 Table Differences of various clinical parameters according to microRNA gene polymorphisms in ischemic stroke patients. Data are mean ± standard deviation and P-value derived by one-way analysis of variance test. aPTT = Activated Partial Thromboplastin Time, BUN = blood urea nitrogen.
(PDF)
