# Supplementary material for: Association of the Single Nucleotide Polymorphisms in microRNAs 130b, 200b, and 495 with Ischemic Stroke Susceptibility and Post-Stroke Mortality
Source: PLoS One. 2016 Sep 7;11(9):e0162519. doi: 10.1371/journal.pone.0162519 (PMC5014326; doi:10.1371/journal.pone.0162519)
Supplement: S2 Table — Data are presented as AOR (95% CI) and P-value derived from multivariate logistic regression. Adjustments were performed for age, sex, hypertension, diabetes mellitus, hyperlipidemia, and current smoking. AOR = adjusted odds ratio for ischemic stroke, 95% CI = 95% confidence interval. (PDF) [file pone.0162519.s002.pdf]

S2 Table. Association of microRNA genotypes with ischemic stroke susceptibility in subgroup analysis considering individual risk factors

Risk factor	Subgroups	***miR-130b* rs373001 TC+CC**		***miR-200b* rs7549819 TC+CC**		***miR-495* rs2281611 AC+CC**	
		**AOR (95% CI)**	***P***	**AOR (95% CI)**	***P***	**AOR (95% CI)**	***P***
Age	<63	1.181 (0.775 – 1.802)	0.439	0.975 (0.640 – 1.486)	0.906	1.676 (1.011 – 2.779)	0.045
	≥63	0.958 (0.664 – 1.381)	0.817	0.883 (0.613 – 1.273)	0.505	0.825 (0.522 – 1.305)	0.411
Sex	Male	1.296 (0.842 – 1.995)	0.238	1.047 (0.684 – 1.602)	0.834	1.109 (0.649 – 1.895)	0.705
	Female	0.906 (0.631 – 1.301)	0.593	0.870 (0.604 – 1.252)	0.452	1.110 (0.715 – 1.721)	0.642
Hypertension	No	1.021 (0.681 – 1.532)	0.920	0.953 (0.636 – 1.428)	0.816	1.122 (0.693 – 1.819)	0.639
	Yes	1.068 (0.732 – 1.558)	0.733	0.880 (0.603 – 1.285)	0.508	1.129 (0.703 – 1.813)	0.615
Diabetes mellitus	No	1.061 (0.781 – 1.442)	0.703	0.927 (0.683 – 1.259)	0.628	1.182 (0.814 – 1.718)	0.380
	Yes	0.965 (0.512 – 1.818)	0.911	0.862 (0.453 – 1.639)	0.650	0.932 (0.417 – 2.081)	0.863
Hyperlipidemia	No	1.102 (0.803 – 1.512)	0.549	0.989 (0.721 – 1.357)	0.945	1.107 (0.746 – 1.643)	0.614
	Yes	0.867 (0.495 – 1.519)	0.617	0.759 (0.431 – 1.338)	0.340	1.137 (0.589 – 2.195)	0.701
Smoking	No	0.886 (0.628 – 1.249)	0.489	0.830 (0.588 – 1.171)	0.288	1.102 (0.720 – 1.686)	0.655
	Yes	1.457 (0.909 – 2.335)	0.118	1.160 (0.731 – 1.841)	0.530	1.117 (0.636 – 1.959)	0.701

Data are AOR (95%) CI and P-value derived by multivariate logistic regression.

Adjustments were performed for age, sex, hypertension, diabetes mellitus, hyperlipidemia and smoking.
AOR = adjusted odds ratio for ischemic stroke, 95% CI = 95% confidence interval.
