# Supplementary material for: Association of the Single Nucleotide Polymorphisms in microRNAs 130b, 200b, and 495 with Ischemic Stroke Susceptibility and Post-Stroke Mortality
Source: PLoS One. 2016 Sep 7;11(9):e0162519. doi: 10.1371/journal.pone.0162519 (PMC5014326; doi:10.1371/journal.pone.0162519)
Supplement: S3 Table — Data are as mean ± standard deviation and P-value derived from a one-way analysis of variance test. PLT = platelet count, PT = prothrombin time. (PDF) [file pone.0162519.s003.pdf]

S3 Table. Differences of various clinical parameters according to microRNA gene polymorphisms in ischemic stroke patients

Characteristics	Homocysteine (mmol/l)	Folate (mg/ml)	Vitamin B12 (pg/ml)	Total cholesterol (mg/dl)	Triglyceride (mg/dl)	PLT ($x10^3/\mu l$)
***miR-130b*T>C rs373001**						
TT	10.87 ± 5.26	7.52 ± 5.79	755.20 ± 728.92	191.97 ± 41.38	146.13 ± 89.94	243.24 ± 70.24
TC	10.79 ± 6.51	8.08 ± 7.87	736.08 ± 552.26	189.32 ± 36.94	155.72 ± 123.00	245.02 ± 66.17
CC	10.17 ± 5.11	8.41 ± 5.89	776.77 ± 648.31	192.94 ± 43.18	146.39 ± 107.78	267.43 ± 157.75
P	0.637	0.348	0.865	0.594	0.416	0.449
***miR-200b*T>C rs7549819**						
TT	10.73 ± 5.22	8.08 ± 8.09	729.00 ± 685.05	192.33 ± 40.08	148.45 ± 106.16	248.48 ± 79.53
TC	10.61 ± 4.81	7.68 ± 5.32	785.56 ± 689.36	190.33 ± 39.58	149.45 ± 103.61	242.65 ± 82.06
CC	11.78 ± 9.88	7.06 ± 4.63	686.25 ± 401.05	188.94 ± 41.13	155.57 ± 102.16	246.90 ± 60.36
P	0.515	0.358	0.288	0.665	0.834	0.565
***miR-495*A>C rs2281611**						
AA	10.57 ± 7.08	7.65 ± 5.64	696.44 ± 397.11	194.73 ± 42.98	148.66 ± 95.47	244.01 ± 74.87
AC	10.84 ± 5.44	7.95 ± 6.16	761.48 ± 692.05	190.03 ± 39.66	154.22 ± 118.16	244.07 ± 84.50
CC	10.90 ± 4.78	7.61 ± 8.34	778.44 ± 797.71	189.66 ± 37.25	140.84 ± 78.48	250.82 ± 70.00
P	0.806	0.771	0.370	0.296	0.211	0.539

Data are mean ± standard deviation and P-value derived by one-way analysis of variance test. PLT = platelet count, PT = prothrombin time
