# Supplementary material for: Association of the Single Nucleotide Polymorphisms in microRNAs 130b, 200b, and 495 with Ischemic Stroke Susceptibility and Post-Stroke Mortality
Source: PLoS One. 2016 Sep 7;11(9):e0162519. doi: 10.1371/journal.pone.0162519 (PMC5014326; doi:10.1371/journal.pone.0162519)
Supplement: S4 Table — Data are mean ± standard deviation and P-value derived by one-way analysis of variance test. aPTT = Activated Partial Thromboplastin Time, BUN = blood urea nitrogen. (PDF) [file pone.0162519.s004.pdf]

S4 Table. Differences of various clinical parameters according to microRNA gene polymorphisms in ischemic stroke patients

Characteristics	PT (sec)	aPTT (sec)	Fibrinogen (mg/dl)	Antithrombin (%)	BUN (mg/dl)	Uric acid (mg/dl)
***miR-130b*T>C rs373001**						
TT	11.94 ± 3.42	31.55 ± 8.51	412.97 ± 134.35	92.86 ± 30.30	16.07 ± 7.22	4.66 ± 1.49
TC	11.77 ± 1.14	31.55 ± 16.36	437.72 ± 122.54	94.95 ± 16.10	16.26 ± 6.10	4.69 ± 1.64
CC	11.86 ± 0.87	31.42 ± 4.33	436.02 ± 129.38	91.98 ± 25.12	16.09 ± 4.69	4.62 ± 1.43
P	0.718	0.997	0.069	0.588	0.922	0.949
***miR-200b*T>C rs7549819**						
TT	11.78 ± 1.05	30.91 ± 5.79	424.51 ± 128.67	95.48 ± 34.10	16.27 ± 7.53	4.69 ± 1.53
TC	11.99 ± 3.77	31.49 ± 8.72	421.99 ± 131.64	92.34 ± 16.94	16.05 ± 5.91	4.68 ± 1.55
CC	11.75 ± 0.93	34.35 ± 29.15	426.22 ± 132.58	90.57 ± 14.49	15.97 ± 5.74	4.54 ± 1.56
P	0.535	0.348	0.960	0.221	0.861	0.699
***miR-495*A>C rs2281611**						
AA	12.14 ± 5.21	31.26 ± 9.49	413.58 ± 115.48	93.65 ± 15.73	16.03 ± 4.65	4.44 ± 1.44
AC	11.86 ± 1.03	31.84 ± 14.37	423.02 ± 135.79	93.28 ± 31.53	16.53 ± 8.02	4.74 ± 1.62
CC	11.63 ± 0.79	31.13 ± 6.29	434.06 ± 130.94	93.96 ± 18.60	15.41 ± 4.93	4.74 ± 1.44
P	0.172	0.741	0.410	0.963	0.076	0.034

Data are mean ± standard deviation and P-value derived by one-way analysis of variance test. aPTT = Activated Partial Thromboplastin Time, BUN = blood urea nitrogen.
